# Oral immunotherapy combined with omalizumab for high–risk cow’s milk allergy: a randomized controlled trial

**DOI:** 10.1038/s41598-017-16730-6

**Published:** 2017-12-12

**Authors:** Masaya Takahashi, Kazuhiko Soejima, Shoichiro Taniuchi, Yasuko Hatano, Sohsaku Yamanouchi, Hideki Ishikawa, Makoto Irahara, Youhei Sasaki, Hiroshi Kido, Kazunari Kaneko

**Affiliations:** 1 Department of Pediatrics, Kansai Medical University, Osaka, 573-1191 Japan; 2 Department of Molecular-Targeting Cancer Prevention, Graduate School of Medical Science, Kyoto Prefectural University of Medicine, Kyoto, 602-8566 Japan; 3 Department of Pediatrics, Institute of Biomedical Sciences, Tokushima University Graduate School, Tokushima, 770-8501 Japan; 4 Division of Enzyme Chemistry, Institute for Enzyme Research, Tokushima University, Tokushima, 770-8501 Japan; 5 Department of Pediatrics, Takatsuki General Hospital, Osaka, 569-1192 Japan

## Abstract

We evaluated the efficacy and safety of oral immunotherapy (OIT) combined with 24 weeks of omalizumab (OMB) at inducing desensitization in children with cow’s milk allergy (CM) compared with an untreated group. The present study was a prospective randomized controlled trial. Sixteen patients (age, 6–14 years) with high IgE levels to CM were enrolled in the present study. Patients were randomized 1:1 to receive OMB-OIT group or untreated group. The primary outcome was the induction of desensitization at 8 weeks after OMB was discontinued in OMB-OIT treated group and at 32 weeks after study entry. None of the 6 children in the untreated group developed desensitization to CM while all of the 10 children in the OIT-OMB treated group achieved desensitization (P < 0.001). A significantly decreased wheal diameter in response to a skin prick test using CM was found in the OMB-OIT treated group (P < 0.05). These data suggest that OIT combined with OMB using microwave heated CM may help to induce desensitization for children with high-risk CM allergy. This prospective randomized controlled trial was intended for 50 participants but was prematurely discontinued due to overwhelming superiority of OMB combined with microwave heated OIT over CM avoidance.

## Introduction

Allergy to cow’s milk (CM) is the second most common immediate-type food allergy in Japanese children^1^. Allergen avoidance is the basic approach for the management of food allergy until clinical tolerance is introduced. Approximately 50% of children can tolerate CM by 5 years of age, increasing to 75% by their early teenage years^2^. Nevertheless, some children experience persistent allergic reactions^3,4^.

Oral immunotherapy (OIT) for food allergy has been used often for young children with CM allergy and has been shown to be effective in several studies^5–15^. However, adverse effects occur frequently during OIT (especially during the escalation phase) and use of parenteral epinephrine is not infrequent. As many as 20–30% of patients with food allergy are refractory to desensitization, particularly those with higher initial food specific immunoglobulin E (sIgE) levels^16,17^. In general, patients with severe asthma can be treated with the anti-IgE monoclonal antibody omalizumab (OMB)^18–20^. Only three studies using OIT combined with OMB for CM allergy are currently available^21–23^, there are not sufficient to assess the efficacy and safety such combination treatment.

The aim of the present study was to evaluate the efficacy in the introduction of desensitization and safety of OIT combined with OMB for 24 weeks in children with high-risk CM allergy compared with an untreated group. We also evaluated the immunological mechanisms of OMB-OIT to measure immunoglobulin (Ig) G subclasses and IgA for CM-related allergens.

## Results

### Study planning

Initially, we planned for 50 children to enter the study. However, when 16 children entered the study, it became difficult to recruit the participants because the first 6 children in the OMB-OIT-treated group passed the double-blind placebo controlled food challenge (DBPCFC) and 5 children in the untreated group did not pass DBPCFC 32 weeks from the start of the study. Therefore, we stopped this trial prematurely because of an ethical decision made by all authors: this trial was not blinded.

### Study population

Eighteen patients were enrolled for eligibility and sixteen patients (age, 6–14 years) were randomized between November 2014 and May 2015. Registered patients ware also assessed between November 2014 and February 2016. No patient was withdrawn after the randomization from the study. Baseline characteristics of these subjects are shown in Table 1. Significant differences were not observed between the two groups for any baseline characteristics (all given as median values): age (OMB-OIT treated group *vs* untreated group, 9.5 *vs* 9.5 years): asthma; other food allergies; history of anaphylaxis; history of atopic dermatitis; total IgE (1993 *vs* 1852 IU/mL); CM-sIgE (85 *vs* 60.9 kUA/L); casein sIgE (97.0 *vs* 52.3 kUA/L); β-lactoglobulin sIgE (16.2 *vs* 12.8 kUA/L); α-lactoalbumin sIgE (3.7 *vs* 2.0 kUA/L); skin prick test (SPT) response to fresh CM (wheal diameter, 11.3 *vs* 15 mm); SPT response to microwave heated CM (MH-CM) (wheal diameter, 12.5 *vs* 9.3 mm); SPT response to standard CM (wheal diameter, 11 *vs* 16 mm); histamine response (wheal diameter, 7.25 *vs* 7 mm); DBPCFC eliciting dose (successfully consumed dose (SCD), 43 *vs* 0 mg); open food challenge (OFC) using fresh CM (1.8 *vs* 0.6 mL); OFC using MH-CM (1.8 *vs* 1.8 mL). Significant differences were not observed between the two groups for any baseline level of IgG subclass (CM, casein, β-lactoglobulin) or IgA characteristics (all given as median values)(data not shown): CM-sIgG1 (1385 *vs* 792 BUg1/mL); casein-sIgG1 (1729 *vs* 941 BUg1/mL); β-lactoglobulin-sIgG1 (193 *vs* 145 BUg1/mL); CM-sIgG2 (330 *vs* 293 BUg2/mL); casein-sIgG2 (361 *vs* 371 BUg2/mL); β-lactoglobulin-sIG2 (80 *vs* 85 BUg2/mL), CM-sIgG3 (50 *vs* 50 BUg3/mL); casein-sIgG3 (50 *vs* 50 BUg3/mL); β-lactoglobulin-sIG3 (50 *vs* 50 BUg3/mL); CM-sIgG4 (89 *vs* 75 BUg4/mL); casein-sIgG4 (89 *vs* 69 BUg4/mL); β-lactoglobulin-sIG4 (51 *vs* 50 BUg4/mL).

**Table 1:** Baseline characteristics of patients in the study groups.

Characteristic	Group	
	OMB-OIT treated (N = 10)	Untreated (N = 6)
Gender (male)	5 (50%)	6 (100%)
**Age at the challenge test**		
Median (Range)	9.5 (8.3–10)	9.5 (7.5–10.8)
**Initial total IgE level (IU/mL)**		
Median (IQR)	1993 (1198–4117.5)	1852.5 (635.2–2895.8)
**Initial CM-specific IgE level (kUA/L)**		
Median (IQR)	85 (43.5–151)	60.9(30.8-92.1)
**Initial Casein, β-lact, α-lact, -specific IgE level**		
Casein, Median (IQR, kUA/L)	97.0 (48.5–140.5)	52.3 (39.5-183)
β-lact, Median (IQR, kUA/L)	16.2 (7.5–38.5)	12.8 (2.8–43.7)
α-lact, Median (IQR, kUA/L)	3.7 (1.7–8.6)	2.0 (1-25.8)
**Wheal diameter of SPT (mm)**		
Fresh CM Median (IQR)	11.3 (10.3–14.8)	15 (9.5–21.3)
MH-CM Median (IQR)	12.5 (7–16.5)	9.3 (8.6–18.9)
Standard CM exact Median (IQR)	11 (9.6–14.8)	16 (9.5–22.1)
Histamine Median (IQR)	7.3 (6–7.9)	7 (4.5-8.1)
**Presence of Other Food Allergies**		
No	10 (%)	0
Yes	90 (%)	6 (100%)
**Frequency of anaphylaxis at accidental ingestion**		
Never	1 (10%)	1 (17%)
Once	5 (50%)	1 (17%)
2-10 times	3 (30%)	4 (66%)
>10 times	1 (10%)	0
Atopic dermatitis	6 (60%)	3 (50%)
**Asthma**		
Severity of asthma*	3 (30%)	4 (66%)
Intermittent	3 (30%)	3 (50%)
Moderate	0	1 (16%)
Persistent	0	0
SCD of CMP at DBPCFC (mg), median (IQR)	43 (10.8–43)	0 (0–32.5)
SCD of CM at fresh OFC (mL), median (IQR)	1.8 (0.8–1.8)	0.6 (0.3–0.8)
SCD of CM at MH-OFC (mL), median (IQR)	1.8 (0.8–3.3)	1.8 (0.8–3.3)

OMB: omalizumab, OIT: oral immunotherapy, β-lact: β-lactoglobulin, α-lact: α-lactoalbumin, MH: microwave heated, CM: cow’s milk, OFC: open food challenge, ^#^Grade of anaphylaxis according to Sampson’s grading score^24^, DBPCFC: double-blind placebo controlled food challenge, IQR: interquartile range, CMP: cow’s milk protein, SCD: successfully comsumed dose, *Severity of asthma according to Japanese Pediatric Guideline for the treatment and management of bronchial asthma^37^.

### Clinical outcomes

**Figure 1:**
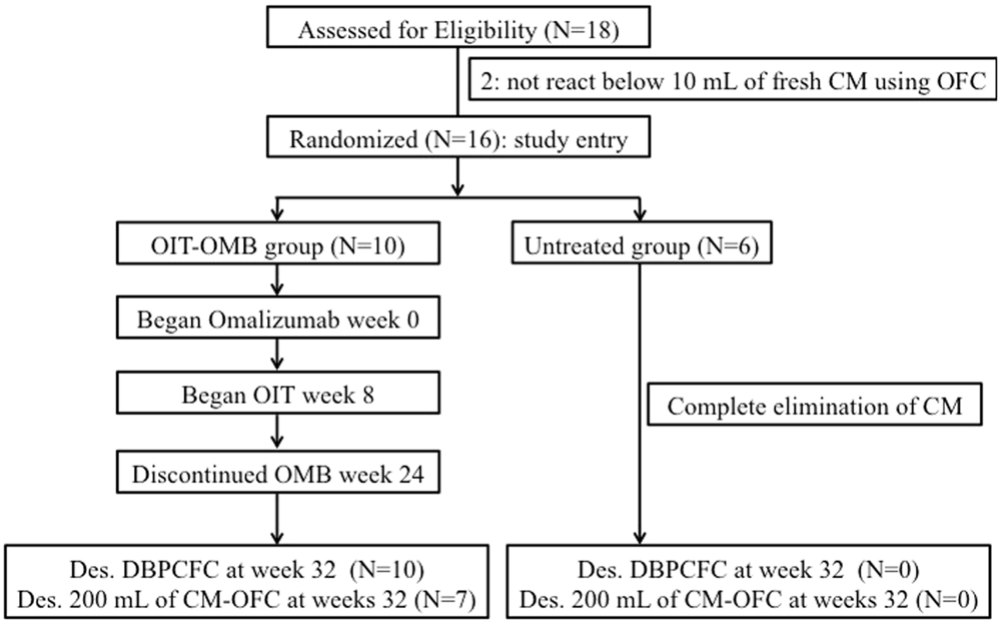
Consort diagram of participant flow in the oral immunotherapy (OIT) with cow’s milk (CM) combined with omalizumab (OMB). Both double-blind controlled placebo food challenge (DBPCFC) and open food challenge (OFC) were perfomed at weeks 32 (last day of the study) to assess desensitization (Des.).

All patients in the OMB-OIT treated group could tolerate 200 mL of fresh CM for a median of 17 (range, 16–21) days without serious adverse events in the escalation phase. Study enrollment and outcomes are shown in Fig. 1. At weeks 32, all OMB-OIT treated patients and none of the untreated patients passed DBPCFC (P < 0.001, Table 2). Also 7 of 10 OMB-OIT treated patients and none of untreated patients passed the OFC using 200 mL of fresh CM (P = 0.01136 , Table 2). In DBPCFC, SCD (median, 2080 mg; range, 2080–2080 mg) in the OMB-OIT treated group was significantly higher than that in the untreated group (0 mg; 0–130 mg) (P < 0.001) and no allergic symptom was noted in the OMB-OIT group compared with the untreated group (P < 0.001). In the OMB-OIT treated group, a significant high SCD for fresh CM (median, 200 mL; range, 60–200 mL) was found compared with that in the untreated group (0; 0–0) (P = 0.006), but no significant difference in the prevalence of allergic reactions in both groups was found.

**Table 2:** The results of desensitization in the two groups at 32 week of the study entry.

Characteristics	Group		*p* value
	OIT (N = 10)	Untreated (N = 6)	
Passed DBPCFC	10/10	0/6	<0.001
**SCD of CMP at DBPCFC (mg)**			
0	0	5	<0.001
43	0	0	
130	0	1	
303	0	0	
650	0	0	
1340	0	0	
2080	10	0	
**Grade of allergic reaction at DBPCFC** ^**#**^			
0	10	0	<0.001
1	0	3	
2	0	3	
3	0	0	
4	0	0	
Passed fresh CM OFC	7/10	0/6	0.01136
**SCD of CM at OFC (mL)**			
0	0	6	0.006
15	0	0	
45	0	0	
105	3	0	
200	7	0	
**Grade of allergic reaction at DBPCFC** ^**#**^			
0	7	0	0.075
1	0	0	
2	1	6	
3	2	0	
4	0	0	

^#^Grade of anaphylaxis according to Sampson’s grading score^24^ OIT: oral immunotherapy, SCD: successfully consumed dose, CMP: cow’s milk protein, DBPCFC: double-blind placebo controlled food challenge, CM: cow’s milk, OFC: open food challenge, IQR: interquartile range, CS: casein, β-lact: β-lactoglobulin, α-lact: α-lactoalbumin.

### Immunologic responses and SPTs

There was no significant difference in levels of total IgE, CM sIgE, casein sIgE, β-lactoglobulin sIgE, or α-lactoalbumin sIgE between the two groups at weeks 32. However, based on median value, the wheal diameter for fresh CM (9.8 mm; interquartile range, 9–11.1 mm), MH-CM (9; 7–9.4) and standard CM extract (8.5; 7–11.3) in response to the SPT in the OMB-OIT treated group was significantly smaller than that in fresh CM (13.5; 11–14.9), MH-CM (13; 11.8–13.9) and standard CM extract (13.3; 7.5–35) in response to the SPT in the untreated group (Table 3). In both groups, there was no significant difference in levels of total IgE or sIgE in response to CM, casein, β-lactoglobulin, or α-lactoalbumin (Table 3). In the OMB-OIT treated group, the wheal diameter in response to each SPT decreased gradually in a time-dependent manner (data not shown). A significant reduction in wheal diameter was noted at weeks 16, 24, and 32 compared with that at baseline for a standard SPT (data not shown). For SPTs to fresh CM and MH-CM, a significantly reduced wheal diameter was found at all stages (data not shown). In the untreated group, no significant change in wheal diameter was found at any stage from baseline (data not shown).

**Table 3:** The results of total IgE, CM, CS, β-lact, α-lact-specific IgE in the two groups at 32 week of the study entry.

Characteristics	Group		*p* value
	OIT (N = 10)	Untreated (N = 6)	
Total IgE level (IU/mL) Median (IQR)	2793 (1370–5160)	2060 (1015–4126)	0.448
CM-specific IgE level (kUA/L) Median (IRQ)	218 (68–348)	40 (32–90)	0.159
**CS, β-lact, α-lact, -specific IgE level (kUA/L)**			
Casein, Median (IRQ)	210(72–409)	42 (35–105)	0.083
β-lact, Median (IRQ)	36 (16–58)	3 (2–8)	0.083
α-lact, Median (IRQ)	8 (2–28)	8 (1–17)	0.448
**Wheal diameter of SPT (mm)**			
Fresh CM Median (IRQ)	9.8 (9–11.1)	13.5 (11–14.9)	0.033
MH-CM Median (IRQ)	9 (7–9.4)	13 (11.8–13.9)	0.003
Standard CM exact Median (IRQ)	8.5 (7–11.3)	13.3 (7.5–35)	0.022
Histamine Median (IRQ)	6.8 (6.5–7.8)	4.8 (4.5–5.8)	0.014

CM: cow’s milk, IQR: interquartile range, CS: casein, β-lact: β-lactoglobulin, α-lact: α-lactoalbumin, MH: microwave heated.

Based on median values, the level for IgG1-CM (4186 BUg1/mL; interquartile range, 2956–5555 BUg1/mL), IgG1-casein (4420; 2822–5676), IgG1-β-lactoglobulin (1163; 662–1646), IgG2-CM (641 BUg2/mL; interquartile range 483–7937 BUg2/mL), IgG2-β-lactoglobulin (289; 186–341) and IgG4-β-lactoglobulin (110 BUg4/mL; interquartile range, 58–252 BUg4/mL) in the OMB-OIT treated group was significantly higher than that for IgG1-CM (934 BUg1/mL; interquartile range, 580–1589 BUg1/mL), IgG1-casein (1199; 758–1861) and IgG1-β-lactoglobulin (146; 126–245), IgG2-CM (302 BUg2/mL; 231–457 BUg2/mL), IgG2-β-lactoglobulin (83; 80–132) and IgG4-β-lactoglobulin (50 BUg4/mL; interquartile range, 50–51 BUg4/mL) in the untreated group.

Dynamic changes in CM related allergens specific for IgG1, IgG2, IgG4 and IgA were observed during combination therapy, whereas those of allergens specific for IgG1-4 and IgA in the untreated group were not observed (Tables 4 and 5). Serum levels of CM-related allergens specific for IgG2 and IgG4 increased gradually after the rush phase at week 12. Conversely, serum levels of CM-related allergens specific for IgG1 and IgA increased rapidly and significantly at the rush phase at week 12 and decrease gradually during the maintenance phase (Table 4).

**Table 4:** The changes of CM, β-lact, Casein specific IgG1-4 and IgA in the OIT group.

Characteristics	OIT Group					
	BL	W8	W12	W16	W24	W32
**CM-specific**						
IgG1 level (BUg1/mL)	1631 (998–2397)	2004 (909–2262)	4342** (3308–5844)	3752** (2328–4581)	3870** (3397–4963)	4186** (2957–5555)
IgG2 level (BUg2/mL)	330 (279–482)	416 (317–5049	565** (518–663)	572** (546–720)	620** (582–787)	641** (520–919)
IgG3 level (BUg3/mL)	50 (50–50)	50 (50–50)	54 (50–55)	50 (50–57)	51 (50-54)	50 (50–60)
IgG4 level (BUg4/mL)	89 (50–144)	93 (50–120)	167 (68–382)	186* (88–372)	190* (66-241)	184* (88–315)
IgA level (BUa/mL)	23 (14–46)	15 (13-18)	60* (50–60)	47** (21–217)	38 (17–152)	36 (24–106)
**β-lact-specific**						
IgG1 level (BUg1/mL)	193 (172–199)	206 (143–252)	1451** (426–1743)	1590** (440–1879)	943** (402–2040)	1163 (662–1646)
IgG2 level (BUg2/mL)	81 (78–89	91 (67–105)	315** (171–349	345** (194–383	338** (185–367)	289** (186–341)
IgG3 level (BUg3/mL)	50 (50–50)	50 (50–50)	50 (50–50)	50 (50–50)	50 (50–50)	50 (50–50)
IgG4 level (BUg4/mL)	51 (51–54)	50 (50–57)	80 (50–131)	67* (52–211)*	90* (52–211)	110** (58–252)
IgA level (BUa/mL)	12 (10–24)	10 (9–14)	25 (17–30)	23* (11–69)	19 (10–31)	18 (13–30)
**Casein-specific**						
IgG1 level (BUg1/mL)	1729 (1205–2813)	2311 (1199–2480)	4701** (3564–5685)	3800** (2533–5072)	3811** (3475–5482)	4420** (2822–5676)
IgG2 level (BUg2/mL)	361 (291–490)	401 (326–515)	575* (494–673)	597* (483–702)	681* (537–757)	632* (466–911)
IgG3 level (BUg3/mL)	50 (50–50)	50 (50–50)	50 (50–76)	50 (50–53)	50 (50–58)	50 (50–68)
IgG4 level (BUg4/mL)	89 (58–154)	92 (50–129)	189* (70–228)	124 (76–228)	237* ((80–258)	161* (80–270)
IgA level (BUa/mL)	23 (13–36)	15 (13–18)	87** (62–116	58** (25–253)	47 (22–204)	50* (26–140

OIT: oral immunotherapy, CM: cow’s milk, β-lact.: β-lactoglobulin, Each data was express as Median (IQR), transformed as log distribution and that at weeks 8 (W8), 12 (W12), 16 (W16), 24 (W24), and 32 (W32) was compared with that at baseline (BL). *P < 0.05 **P < 0.01.

**Table 5:** The changes of CM, β-lact, Casein specific IgG1-4 and IgA in the untreated groups.

Characteristics	Untreated Group				
	BL	W8	W16	W24	W32
**CM-specific**					
IgG1 level (BUg1/mL)	792 (515–1392)	760 (648–1738)	934 (708–2031)	996 (844–2663)	934 (580–1589)
IgG2 level (BUg2/mL)	293 (194–446)	286 (247–435)	339 (272–484)	362 (338–438)	302 (231–457)
IgG3 level (BUg3/mL)	50 (50–50)	50 (50–50)	50 (50–50)	50 (50–50)	50 (50–50)
IgG4 level (BUg4/mL)	75 (58–78)	68 (59–94)	70 (68–111)	67 (52–211)	90 (52–211)
**β-lac-specific**					
IgG1 level (BUg1/mL)	145 (115–251)	132 (116–252)	161 (120–312)	151 (130–287)	146 (122–245)
IgG2 level (BUg2/mL)	85 70–150)	86 (68–142)	76 (74–159)	98 (73–102)	83 (80–132)
IgG3 level (BUg3/mL)	50 (50–50)	50 (50–50)	50 (50–50)	50 (50–50)	50 (50–50)
IgG4 level (BUg4/mL)	50 (50–50)	50 (50–50)	51 (50–52)	50 (50–51)	50 (50–51)
IgA level (BUa/mL)	21 (13–30)	23 (11–28)	29 (11–38)	22 (8–29)	25 (11–40)
**Casein-specific**					
IgG1 level (BUg1/mL)	941 (653–1786)	1018 (838–2034)	1029 (1002–2309)	1124 (1080–3020)	1199 (758–1861)
IgG2 level (BUg2/mL)	371 (241–462)	389 (276–464)	396 (287–559)	409 (403–466)	397 (288–504)
IgG3 level (BUg3/mL)	50 (50–50)	50 (50–53)	50 (50–52)	50 (50–50)	50 (50–51)
IgG4 level (BUg4/mL)	77 (61–86)	66 (64–92)	76 (74–116)	77 (55–101)	86 (66–97)
IgA level (BUa/mL)	22 (14–34)	21 (12–32)	28 (13–50)	21 (9–39)	29 (11–42)

CM: cow’s milk, β-lact: β-lactoglobulin Each data was expressed as median (IQR), transformed as log distrubution and that at weeks 8 (W8), 16 (W16), 24 (W24), and 32 (W32), those was compared with that at baseline (BL).

### Safety data during OIT

Table 6 shows the average frequencies of adverse reactions that occurred per dose in each patient at hospital or at home. In the escalation phase of OIT (at hospital), a total of 0.012 adverse reactions per dose per child occurred. According to Sampson’s grading score^24^, there were 0.03 grade-1 adverse reactions, 0.007 grade-2 adverse reactions, and no grade-3 or grade-4 adverse reactions. At home during the following year, a total of 0.048 adverse events per dose per child occurred. Also, there were 0.016 grade-1 adverse reactions, 0.033 grade-2 adverse reactions, 0.01 grade-3 adverse reactions, and no grade-4 adverse reactions.

**Table 6:** Averages of adverse reactions and treatments per dose per child in the escalation phase (at hospital), and maintenance phase of oral immunotherapy.

Phase	Total	Grade 1^#^	Grade 2^#^	Grade 3^#^	Grade 4^#^	
Escalation (401 total doses)	0.012	0.003	0.007	0	0	
Maintenance (1414 total doses)	0.028	0.004	0.021	0.002	0	
**Phase**	**Anti- histamines**	**Nebulized epinephrine**	**Nebulized β**_**2**_ **agonists**	**Oral steroids**	**Intravenous steroids**	**Epinephrine injections**
Escalation	0.020	0.039	0	0	0	0
Maintenance	0	0	0.013	0.013	0	0

Reactions per dose per child. Each data express averages of reactions and treatments per dose per child. ^#^Grade of anaphylaxis according to Sampson’s grading score^24^.

For treatment of these symptoms, 2 of 10 (20%) children and 4 of 10 children (40%) received medication at hospital and at home, respectively. Mean numbers of treatment per dose per child are shown in Table 3. No epinephrine injections were given in either phase.

Anaphylactic shock and death induced by CM ingestion were not observed in either group during the study.

### Patient characteristics

Characteristics of 10 children in the OMB-OIT treated group are described in detail in Table 7. All children passed DBPCFC and 7 of 10 children tolerated 200 mL of fresh CM for the OFC. Levels of total IgE at baseline in 6 of 10 children were >1500 IU/mL. These 6 children passed DBPCFC, and 5 of the 6 children passed a 200 mL OFC.

**Table 7:** Patient’s characteristics of the OIT-OMB treated group.

No	G	Age	History of LTE	SCD** at baseline (mL)	SCD** at weeks 8 (mL)	SCD** at weeks 32 (mL)	Total IgE (IU/mL)	CM- sIgE (kUA/L)	OMB (mg)
1	F	13	No	1.8	87	200	5164	54.4	525
2	M	9	No	0.8	88	200	1152	24	300
3	M	7	Yes (2)*	1.8	17	200	4316	157	300
4	F	8	Yes (1)*	0.8	0	45	1336	2040	375
5	F	10	No	3.8	15	200	42990	75	375
6	M	12	Yes (1)*	3.8	35	105	1553	38.9	375
7	M	9	Yes (1)*	1.8	37	105	578	95	300
8	M	10	No	0	18.8	200	3521	307	375
9	F	10	Yes (3)*	0.8	38	200	2433	40.2	375
10	F	8	No	1.8	37	200	1024	133	300

OIT: oral immunotherapy, OMB: omalizumab, G: gender, LTE: life threatening event, SCD: successfully consume dose, CM-sIgE: cow’s milk specific IgE, M: male, F: female, *Frequencies of LTE including hypotension. **Fresh CM open food challenge at baseline, weeks 8 and 32.

Surprisingly, 5 children who experiences anaphylactic shock (which is a life-threatening event) passed DBPCFC and 3 of these 5 children passed a 200 mL OFC.

## Discussion

We planned this randomized study using the CM-avoidance group as a control group for two main reasons. First, the effect of OIT on CM allergy has not been demonstrated completely even though several OIT trials focusing on CM allergy^5–15^ have been completed. Only 5 RCTs have been reported and the effect in those studies has not been demonstrated completely because they were small^25^. Second, several OIT studies^5–15^ had problems regarding patient safety, and frequent severe adverse events during OIT occurred in cases of high-risk CM allergy. Before the beginning of our trial, we planned for 50 participants with high-risk CM allergy to be recruited (as mentioned at the ‘study design’ portion of the Methods section).

Several OITs for IgE-mediated CM allergy have been reported but, in the case of children with high sIgE levels due to CM, efficacy is quite low with frequent adverse events, even if desensitization is successful^16,17^. Our previous study on CM allergy showed that desensitization was achieved only half for a year interval, and that children with sIgE >20 kUA/L to CM at study entry could not achieve desensitization and suffered frequent severe adverse events during OIT^17^. Compared with our previous study^17^, it should be noted that all children were desensitized with mild tolerable allergic events in the present study. Recently, Wood *et al*. in a randomized double-blind placebo-controlled study of OMB combined with OIT for CM allergy^23^, demonstrated significant improvements in safety but not in efficacy. They had a control group that underwent OIT without OMB treatment, but not a control group that had complete elimination of CM. In addition, they excluded patients who had experienced life-threatening events to CM/CM products^23^.

In contrast, the patients with those who experienced life-threatening anaphylaxis to CM/CM products were allocated into the OMB-OIT treated group in the present study: Five of 10 children who underwent treatment with OIT in combination with OMB experienced life-threatening anaphylaxis to CM/CM products. Although all patients who participated in our study were high-risk for anaphylaxis to CM, they are able to achieve desensitization.

In our study, severe adverse events were not observed even during the escalation phase. In terms of alleviating the adverse events during OIT the OMB-OIT treated group, we used MH-CM rather than fresh CM in the present study because: (i) MH-CM can be prepared very easily; (ii) microwave irradiation of CM has been shown to reduce the risk of allergic responses in a mouse model of allergy similar to that observed for conventional heating^26^; (iii) microwave treatments cause peptic hydrolysis to occur quickly, as demonstrated in a murine model of allergy *ex vivo* ^27^. Also, in our previous study^17^, 70% of children who underwent OIT using MH-CH could remain unresponsive in the escalation phase. As CM can be heated very easily and rapidly using microwave oven, and the effect of MH is similar to that of boiling for 30 min^26^, MH-CM is recommended for OIT in children with CM allergy to alleviate the adverse events and to achieve tolerance of CM for long periods. However, an RCT study comparing MH-CM and CM for OIT directly is needed.

Thirty-two weeks after study entry, the wheal diameter in response to SPTs (three types of CM products) in the OMB-OIT treated group was significantly less than that in the untreated group. However, CM-related sIgE levels in the OMB-OIT treated group did not decrease significantly. In general, it is considered that the SPT provides an ‘*in vivo*’ procedure for measuring the reactivity of sIgE antibody-activated mast cells and basophils and in fact, rapid decrease in the number of mast cells and basophils in skin, as determined by SPTs, has been observed in immunotherapy for aeroallergens^28^ and food allergens^6,29^. Meanwhile, the half-life of IgE is 2 days while that of OMB, a humanized IgG antibody, is as long as 21 days. After administration, OMB binds with IgE antibodies, and the levels of various sIgE types including CM-related sIgE increase. Taken together, it appears that sIgE levels do not reflect desensitization in the OMB-OIT treated group and that the SPT is the only way to predict desensitization.

Collins and Jackson^30^ suggested that early in the germinal center reaction, IgM+ B cells switch first to IgA, IgE and IgG3, then to IgG1 cells, followed by IgG2-committed cells and finally, upon continued exposure to the antigen, to IgG4-producing cells, which coincides with the arrangement sequence of the immunoglobulin heavy gene locus. Patients received high doses of an allergen repeatedly during OIT, so the remarkable changes in levels of IgG subclasses observed in the present study can be explained by a class switching pathway: μ → γ3 → γ1 → γ2 → γ4. We demonstrated that rush OIT using eggs induces this immunoglobulin class switching pathway, and that high serum levels of allergen-specific egg-related IgG1 after the rush phase of OIT are potentially suitable biomarkers for positive immune responsiveness to OIT^31^. Recent studies have shown that high levels of sIgE before OIT are less predictive of sustained unresponsiveness^17,32^. OMB administration reduces the level of free IgE markedly, which causes a reduction in the number of Fcε receptors on antigen-presenting cells, mast cells and basophils^33^. This event may induce FoxP3-positive CD4 T regulatory cells^34,35^. These cells produce suppressive cytokines such as TGF-β or Il-10. In general, high-affinity IgE is also generated through sequential class switching (μ → γ3 → γ1 → ε)^36^ in allergic inflammatory states. Collectively, the success of this class switching (μ → γ3 → γ1 → γ2 → γ4) instead of sequential class switching (μ → γ3 → γ1 → ε) may be because OMB administration induces a reduction in the levels of free IgE, followed by a reduction in the number of Fcε receptors of these Th2-related cells. This action causes immunoglobulin class switching by repeated exposure to CM, and results in increased levels of IgG2 and IgG4. This change in class switching was observed in both specific casein and β-lactoglobulin in the present study. It would be need to elucidate whether this change will be due to only CM-OIT or combination CM-OIT and also whether the relevance of sustained unresponsiveness and increased level of IgG4 to CM is significant or not in future study.

Our study had several limitations. First, the study was randomized, but the placebo controls was not blinded and set as only the untreated group. However, 32 weeks after study entry, DBPCFC was done in both groups, which enable valuable statistical analyses. Second, multiple regression analyses were used to identify independent factors to predict tolerance and reactivity. However, the sample size was too small to ascertain if these factors were significant. Third, the OMB-OIT treated group and untreated group were not equivalent in terms of demographics. There was a trend in the OMB-OIT treated group towards increased numbers of entry children (10:6) and more males in the untreated group. A significantly reduced wheal size in the treatment group was obtained compared with that of the untreated group. However, this result may be not ‘truly’ significant because of selection bias: the groups were comparable at baseline but the control group appeared to have a larger SPT response to CM than the treatment group. Finally, the primary endpoint of our study was not sustained unresponsiveness, but only desensitization. We are planning a follow-up study of OMB-OIT using the 16 children mentioned above to investigate sustained unresponsiveness.

## Methods

### Study design

A prospective, randomized, interventional study was performed from November 2014 to March 2016 at the Department of Pediatrics, Kansai Medical University Hospital, Osaka, Japan. The study protocol was registered in UMIN CTR of Japan (UMIN000015545, registered date: 1st November 2014), and approved by the Institutional Ethics Committee of Kansai Medical University (approval number: 1409) on 7th October 2014. The study was conducted according to the principles in the Declaration of Helsinki.

Inclusion criteria were as follows: aged 6–15 years and persistent CM allergy, that was confirmed by 1) positive clinical history of anaphylaxis caused CM or CM products by last two years, 2) Sampson’s symptoms score >grade 2^24^ associated with below 10 mL in OFC using fresh CM, 3) positive DBPCFC to CM, 4) sIgE (CAP-Phadia, Uppsala, Sweden) level to CM >17.5 kUA/L), and 5) positive SPT response (wheal diameter ≥3 mm larger than that elicited by negative control). We ensured that families had adequate information regarding the study and understood the implications of participation. Signatures designating informed consent were obtained. Exclusion criteria were as follows: patients diagnosed as having acute severe illness, severe atopic dermatitis and uncontrolled asthma (baseline FEV_1_ < 80% of predictive value) according to Japanese pediatric guideline for the treatment and management of bronchial asthm^37^.

Fifty participants were set as a goal before study entry. We were not able to calculate the number of participants as a goal setting by statistically means because study or data about OIT combination using OMB for CM allergy at study entry were lacking. Also 50 children with CM allergy wished to participate in our study.

Subjects were randomized 1:1 to receive OIT combined with OMB (OMB-OIT treated group) or complete elimination of CM/CM products (untreated group). Simple computerized randomization was carried out and allocation was by sequentially numbered, opaque sealed envelops by a hospital nurse. OMB doses in the OMB-OIT treated group were started according to a chart based on a formula provided by Genentech (San Francisco, CA, USA) within 1 week after the study entry. If the subjects had total IgE >1500 IU/mL, then they received an OMB dose of 1500 IU/mL/body weight. OMB treatment was for the first 24 weeks of the study at intervals of 2 weeks or 4 weeks.

The escalation phase of OIT in the OMB-OIT treated group was started after the first 8 weeks of OMB treatment. Two series of CM challenge were carried out before the escalation phase using fresh CM and MH-CM to examine the starting doses in the escalation phase. All patients were admitted to Kansai Medical University Hospital. OIT was modified by adherence to our method described previously^17^. OIT comprised of escalation phase and maintenance phase. Patients had undergone the escalation phase followed by the maintenance phase. Daily dosing at home was continued until week 32 of the start of OMB administration. Then, DBPCFC and an OFC using fresh CM was done to desensitize for CM allergy. OMB administration was stopped 24 weeks from treatment commencement.

In the untreated group, complete elimination of CM was continued until week 32. Then, patients could undergo physical examination, blood tests and the skin prick test (SPT) every 8 weeks. Then, DBPCFC and an OFC using fresh CM were done.

### Food challenge

Clinical features of a reaction to CM were investigated via a DBPCFC and an OFC as described previously^17^. DBPCFC was conducted placebo (*katakuriko*, which is fine vegetable starch) or non-fat milk powder (Yotsuba Nyugyou, Sapporo, Japan), with cumulative doses of 6 g milk powder. DBPCFC was based on six doses (43, 87, 173, 347, 693, and 737 mg) of milk protein given at 15-min intervals. Six grams of *katakuriko* was divided into six doses.

The initial challenge dose of CM was set at 0.1 mL and subsequent doses were 0.2, 0.5, 1, 2, 5, 10, 20, and 50 mL every 30 min. We also used MH-CM for the OFC before OIT to define the initial dose of MH-CM OIT. Fresh CM was heated in a microwave oven at 550 W for 100 s and then cooled to room temperature before the OFC. The MH-CM food challenge was undertaken only with the OMB-OIT treated group immediately before the start of OIT. We also carried out the second OFC using fresh CM and MH-CM 8 weeks from the start of OMB treatment before the start of OIT to ascertain the initial dose of OIT.

DBPCFC and the OFC using fresh CM were done 32 weeks after study entry in both groups to confirm desensitization for CM allergy. In the OMB-OIT treated group, the initial dose of fresh CM was set at 15 mL, and then increased to 30, 60, and finally 95 mL (total dose, 200 mL) every 30 min. If the result of the CM-OFC was negative, the patient was defined as being desensitized to CM.

### OIT

The OIT protocol was modified by an OIT protocol that has been reported previously^17^. OIT was done 8 weeks from the start of OMB treatment. The OIT protocol is comprised an escalation phase and maintenance phase.

Briefly, MH-CM was prepared in the same manner as the material administered for the OFC. The initial dose of OIT was set at a sub-threshold dose that was usually one-tenth of the threshold dose determined by the OFC using MH-CM. After the initial dose, the next and subsequent doses were increased approximately 1.5-fold until the threshold dose was reached. After reaching the threshold dose, the next and subsequent doses were increased approximately 1.2-fold. Subsequent doses were administered every 2 h up to four times in 1 day, or until an allergic reaction occurred. The target dose of CM was 200 mL of MH-CM. If there were no further increases in dose because of repeated adverse events, then, escalation of OIT using fresh CM was started after the highest tolerated dose was continued for 3 consecutive days without an allergic reaction. The initial dose of fresh CM was set at 50 mL, and the next and subsequent doses were increased approximately 1.2-fold. Subsequent doses were administered every 2 h up to four times in 1 day, or until an allergic reaction occurred. If the target dose (200 mL of fresh CM) was achieved, dose escalation was terminated and that dose was chosen as the maintenance phase (i.e., the patients were discharged and ingestion of the target dose was continued at home every day to maintain the effect of OIT in the maintenance phase).

After patients in the OMB-OIT treated group had undergone the escalation phase followed by the maintenance phase, OMB was discontinued 24 weeks from its start of administration. In order to ascertain whether the patients were desensitized to CM, DBPCFC and the OFC using fresh CM were undertaken 32 weeks after study entry both in the OIT-OMB treated group and untreated group.

### Laboratory tests

Blood samples and the SPTs were examined before study entry (baseline), at weeks 8, 12, 16, 24, and 32 of the start of OMB administration in the OMB-OIT treated groups. In untreated groups, blood samples and the SPT were examined at baseline, at weeks 8, 16, 24, and 32 of study entry.

### SPT

The standard CM extract and histamine were purchased by Torii industry (Tokyo, Japan). A fresh CM and MH-CM were used with no dilution.

### Allergens specific immunoglobulins by DCP microarray

Blood samples were collected to measure serum levels of CM-related allergens (CM, casein, β-lactoglobulin)-specific immunoglobulins by densently carboxylate protein (DCP) microarray^32^. This method shows high correlation of allergen-specific IgE values (ρ > 0.9–0.85) determined by the UniCAP system for various allergens^38^ and has been validated^39,40^.

### Outcomes

The primary outcome was the efficacy in the induction of desensitization by the OMB-OIT treatment. Desensitization was defined as the absence of dose-limiting symptoms in the DBPCFC and the OFC using fresh CM at weeks 32. Secondary outcome included change in the OFC SCD, incidence of OIT-related adverse reactions, changes of the SPT, total IgE level, CM-related sIgE levels, CM-related sIgGs and CM-related sIgA.

### Statistical Analysis

We statistically evaluated the primary endpoint of the ratio of the desensitized patients in the OMB-OIT treated group (10 children) and the untreated group (6 children) at weeks 32, using a 2-sided alpha level of 0.05, to detect a significant difference between the 2 groups using Fisher’s exact test. The Mann-Whitney U-test was applied to compare the clinical data except antibody levels in each group of patients between baseline values and those at weeks 32. Fisher’s exact test was also used to evaluate between-group differences with regard to achieving desensitization to 200 mL of fresh CM at weeks 32. We also evaluated the differences in continuous variables except antibody levels using the Wilcoxon signed-rank test. We calculated the level of total IgE, sIgE, IgG1-4, and IgA as log distributions. These transformed parameters were analysed at week 32 (W32) between the two groups by the Student’s *t*-test, and these parameters at W8, W12, W16, W24 and W32 were compared with those at baseline using the paired *t*-test.

### Ethical approval

This study was approved by the Institutional Ethics Committee of Kansai Medical University (approval number: 1409) and the parents of each participant gave written informed consent.

## Conclusion

This randomized study demonstrated that OIT combined with OMB using MH-CM may help to induce desensitization for children with high-risk CM allergy.

### Data monitoring

We thank our data-monitoring committee (Medical Research Support, Osaka, Japan) who recorded the clinical laboratory data (which were anonymized during the study**)** of registered patients.

### Transparency

S.T. (guarantor) affirms that this manuscript is an honest, accurate, and transparent account of the study being reported; that no important aspects of the study have been omitted; and that any discrepancies from the study as planned have been explained.

## Electronic supplementary material

Trial Protocol
