# Author Correction: Oral immunotherapy combined with omalizumab for high–risk cow’s milk allergy: a randomized controlled trial

**DOI:** 10.1038/s41598-018-30919-3

**Published:** 2018-08-21

**Authors:** Masaya Takahashi, Kazuhiko Soejima, Shoichiro Taniuchi, Yasuko Hatano, Sohsaku Yamanouchi, Hideki Ishikawa, Makoto Irahara, Youhei Sasaki, Hiroshi Kido, Kazunari Kaneko

**Affiliations:** 1 Department of Pediatrics, Kansai Medical University, Osaka, 573-1191 Japan; 2 Department of Molecular-Targeting Cancer Prevention, Graduate School of Medical Science, Kyoto Prefectural University of Medicine, Kyoto, 602-8566 Japan; 3 Department of Pediatrics, Institute of Biomedical Sciences, Tokushima University Graduate School, Tokushima, 770-8501 Japan; 4 Division of Enzyme Chemistry, Institute for Enzyme Research, Tokushima University, Tokushima, 770-8501 Japan; 5 Department of Pediatrics, Takatsuki General Hospital, Osaka, 569-1192 Japan

Correction to: *Scientific Reports* 10.1038/s41598-017-16730-6, published online 12 December 2017

The Article contains an error in Table 2. The heading:

“Grade of allergic reaction at DBPCFC^#^”

should read:

“Grade of allergic reaction at OFC”.

Additionally, in Table 3, there are errors present in the “Characteristics” column, where “(IQR)” is incorrectly labelled as “(IRQ)”

